# Supplementary material for: Content-rich biological network constructed by mining PubMed abstracts
Source: BMC Bioinformatics. 2004 Oct 8;5:147. doi: 10.1186/1471-2105-5-147 (PMC528731; doi:10.1186/1471-2105-5-147)
Supplement: Additional File 2 — The original results of the above study (non-essential files are deleted to keep the file size under the limit set by BMC bioinformatics). [file 1471-2105-5-147-S2.bz2 › chilibotAdditionalFile2/dip10/209ID8846021E709/html/ITGA2_ITGB1.html]

 


 **ITGA2** and **ITGB1** 
  
Found 69 abstracts in PubMed, retrieved 10.  
 

 What does Google say? 
 PDF only 
| .edu only 

---

**Interactive relationship** (e.g. stimulation, inhibition, etc)

**Non-interactive relationship** (e.g. studied together, co-existance, homology, etc.)

- This platelet aggregation activity was dose dependently inhibited by specific GPIbalpha antibodies, but not by those antibodies against platelet GPIa  [ **ITGA2** ] , GPIIa  [ **ITGB1** ] , GPIIb and GPIIIa.  Ref: 12782089 Toxicon< MedlineTA>Toxicon,
- TEC were treated with TCDD at concentrations of 0.00.0..0 or 10.0 nM or with 100 nM PCB 126,, 5 pentachlorobiphenyl for 3 days, and were then analysed by flow cytometry for expression of surface antigens using monoclonal antibodies against Hassall s bodies TE 8, TE 16 or against surface structures such as CD29  [ **ITGB1** ] , CD49b  [ **ITGA2** ] , CD49e, CD49f, CD51, CD54, CD58, CD61 and CD106.  Ref: 12799776 Arch Toxicol, 2003
- At 14 weeks of culture, more mature CHMCs expressed CD11b, CD11c, CD29  [ **ITGB1** ] , CD49b  [ **ITGA2** ] , CD49c, CD49d, CD49e, CD51, CD61, and CD54 and weakly expressed CD18 and CD11a.  Ref: 11174197 J Allergy Clin Immunol, 2001
- As model system, we used living human umbilical vein endothelial cells HUVEC adhering to laminin coated surfaces and investigated the distribution of the alpha2beta1 CD29  [ **ITGB1** ]  CD49b  [ **ITGA2** ]  integrin at the contact zone of these cells.  Ref: 12521136 Cell Commun Adhes< MedlineTA>Cell Commun Adhes, 2001
- Data obtained are presented in concurrence with Clusters of Thymic Epithelial Staining CTES classification for various monoclonal antibodies recognizing CD antigens CD1, CD1a, CD6, CD9, CD14, CD16, CD29  [ **ITGB1** ] , CD30, CD32, CD44, CD45RB, CD47, CD48, CD49a, CD49b  [ **ITGA2** ] , CD49c, CD49d, CD49e, CD49f, CD51, CD53, CD54, CD56, CD57, CD63, CD85, CD95, CD98, CD102, CD103, CD106, CD109, CD146, CD147, CD148, CD151, CD152, CD158a, CD158b, CD164, CD165, CD166 and for monoclonal antibodies 1B1 G7, A4, BD46, BLTZ, HP1C5, IND.64, M72, WU947 whose specifities are not yet defined.  Ref: 12725440 Ann Anat, 2003
- We found that T h 1 cells derived from ovalbumin OVA specific TCR transgenic DO11.10 mice showed significantly higher levels of VLA 2 CD49b  [ **ITGA2** ]  CD29  [ **ITGB1** ]  expression than T h 2 cells.  Ref: 12750354 Int Immunol, 2003
